# Health Disparities Investigator Development through a Team-Science Pilot Projects Program

**DOI:** 10.3390/ijerph20075336

**Published:** 2023-03-30

**Authors:** Jerris R. Hedges, Dominic C. Chow, Benjamin Fogelgren, Kathryn L. Braun, JoAnn U. Tsark, Susan Ordinado, Marla J. Berry, Richard Yanagihara, Noreen Mokuau

**Affiliations:** 1 Departments of Medicine and Surgery, John A. Burns School of Medicine, University of Hawai‘i at Mānoa, Honolulu, HI 96813, USA; 2 Department of Medicine, John A. Burns School of Medicine, University of Hawai‘i at Mānoa, Honolulu, HI 96813, USA; 3 Department of Anatomy, Biochemistry and Physiology, John A. Burns School of Medicine, University of Hawai‘i at Mānoa, Honolulu, HI 96813, USA; 4 Office of Public Health Studies, Thompson School of Social Work & Public Health, University of Hawai‘i at Mānoa, Honolulu, HI 96822, USA; 5 Research Corporation University of Hawai‘i, John A. Burns School of Medicine, University of Hawai‘i at Mānoa, Honolulu, HI 96822, USA; 6 Pacific Biosciences Research Center, School of Ocean & Earth Science & Technology, University of Hawai‘i at Mānoa, Honolulu, HI 96822, USA; 7 Departments of Pediatrics and Tropical Medicine, Medical Microbiology & Pharmacology, John A. Burns School of Medicine, University of Hawai‘i at Mānoa, Honolulu, HI 96813, USA; 8 Department of Social Work, Thompson School of Social Work & Public Health, University of Hawai‘i at Mānoa, Honolulu, HI 96822, USA

**Keywords:** pilot projects program, team-science, research studio, connectivity, health disparities, clinical and translational research

## Abstract

Profound health disparities are widespread among Native Hawaiians, other Pacific Islanders, and Filipinos in Hawai‘i. Efforts to reduce and eliminate health disparities are limited by a shortage of investigators trained in addressing the genetic, socio economic, and environmental factors that contribute to disparities. In this conference proceedings report from the 2022 RCMI Consortium National Conference, we describe our mentoring program, with an emphasis on community-engaged research. Elements include our encouragement of a team-science, customized Pilot Projects Program (PPP), a Mentoring Bootcamp, and a mentoring support network. During 2017–2022, we received 102 PPP preproposals. Of these, 45 (48%) were invited to submit full proposals, and 22 (19%) were awarded (8 basic biomedical, 7 clinical, 7 behavioral). Eighty-three percent of awards were made to early-career faculty (31% ethnic minority, 72% women). These 22 awards generated 77 related publications; 84 new grants were submitted, of which 31 were awarded with a resultant return on investment of 5.9. From 5 to 11 investigators were supported by PPP awards each year. A robust usage of core services was observed. Our descriptive report (as part of a scientific conference session on RCMI specialized centers) focuses on a mentoring vehicle and shows how it can support early-stage investigators in pursuing careers in health disparities research.

## 1. Introduction

Health disparities in Hawai‘i are substantial, especially among Native Hawaiians (NH), other Pacific Islanders (OPI), and Filipinos [1,2,3]. Compared to other ethnic groups in Hawai‘i, NHOPI and Filipinos suffer disproportionately higher rates of obesity [4,5], hypertension [6,7], diabetes [8,9,10], chronic kidney disease [11], cardiovascular and cerebrovascular diseases [12,13], and breast and lung cancers [14,15,16]. They also have the lowest life expectancy of any other racial and ethnic group in Hawai’i [3,17]. Research efforts to reduce the disparities are limited by a shortage of skilled and well-trained investigators, especially from these ethnic groups [18,19,20,21]. The paucity of NH, OPI, and Filipinos participating in research as investigators [22] follows a shortage of minority faculty members in the biomedical and health-related fields [23], and the limited health disparities research mentoring of these groups. Further, the traditional model of a single-investigator-led research project is increasingly insufficient for addressing the complex social, economic, and environmental challenges that contribute to health disparities. Professional development organized via mentoring networks, individual development plans (IDPs), research education, and training in community engagement combined with access to pilot project funding has been shown to increase success in health disparities research [24,25,26]. At the 2022 Research Centers in Minority Institutions (RCMI) Consortium National Conference, we shared lessons learned in health disparities investigator development by reporting on the successes and challenges of our RCMI Pilot Projects Program (PPP) during 2017–2022 in regard to health disparities research within the State of Hawai‘i. This conference proceedings paper presents our approach and related outcomes.

## 2. Materials and Methods

With support from the National Institute on Minority Health and Health Disparities (NIMHD), the overall long-term goal of the RCMI Specialized Center, known as Ola HAWAII, is to improve minority health and reduce health disparities for those communities in Hawai‘i that experience social and economic disadvantages, limited healthcare access, and poor health outcomes. A centerpiece of our strategic approach to meeting this goal is our team-science PPP, which provides funding for new and early-stage health disparities investigators. We describe how our Center encouraged a team-science approach by providing infrastructure and resources to develop and support transdisciplinary teams of health disparities investigators and community collaborators conducting basic biomedical, behavioral, and clinical research on the causes of health disparities and the most effective interventions to reduce health disparities among the underserved, multiethnic populations in Hawai‘i.

A team-science approach was encouraged via the following methods: (1) utilizing a multidisciplinary and multi-professional investigator team to review and evaluate pilot projects; (2) encouraging the demonstration of community relevance and participation in pilot project development and selection; (3) providing assistance in multidisciplinary and multi-professional investigator team development during Research Infrastructure Core consultations and with other RCMI sites; (4) demonstrating examples of successful team science during Mentoring Bootcamp sessions; and (5) encouraging team-science participation in institutional health science academic promotions. In addition to encouraging team science, other innovative methods were used and described below. We also describe outcome metrics, such as resultant scientific presentations, peer-reviewed publications, extramural funding, and examples of successful pilot projects that resulted in behavioral changes in community participants in the Section 3.

### 2.1. Leveraging the Infrastructure Design

Ola HAWAII used its four cores—Administrative (AC), Research Infrastructure (RIC), Investigator Development (IDC), and Community Engagement (CEC)—for integrated support of the PPP mechanism. The cores were also advised by the CEC Hui, a separate advisory group comprising grassroots community members, minority scientists and physicians, and community-based organization representatives. Innovations introduced by the IDC to aid early-stage investigators included (1) customizing the PPP to prioritize team-science projects and strengthening the mentoring environment of PPP-funded investigators through use of IDPs; (2) instituting a novel Mentoring Bootcamp program with tailored sessions relevant to planning and implementing transdisciplinary team-science projects; and (3) expanding access of new and early-stage investigators to mentors, collaborators, and reviewers outside of Hawai‘i, thus providing connections to training programs, multi-site projects, shared resources, and new team-science opportunities at other RCMI grantee institutions.

### 2.2. Customizing the Pilot Projects Program

Ola HAWAII customized the PPP, selecting meritorious team-science projects and strengthening the mentoring environment of diverse investigators through individualized research and career mentoring plans. The annual PPP application process began with a university-wide call for submission of an initial one-page preproposal, along with the investigator’s NIH biosketch and a letter of support from a senior mentor. These PPP preproposals were independently evaluated by three reviewers—two identified by the IDC with one member from among the core leaders (with related content expertise), and one member from the CEC Hui. Individuals with conflicts of interest, either real or perceived, were recused. Reviewers used the NIH review criteria (significance, innovation, approach, investigator, environment) plus three Ola HAWAII-specific criteria (alignment with the health disparities mission, collaborations and partnerships, and use of the Center’s cores). Using the NIH scoring scale (1 = exceptional to 9 = poor), preproposals were scored and ranked (based on reviewer scores) prior to evaluation by Ola HAWAII leadership to prioritize invitations to submit full PPP proposals. Reviewer comments were shared with applicants to help guide the applicant in preparing their full proposals or other future proposals.

Full proposals were independently reviewed by two scientific reviewers with content expertise from outside Ola HAWAII, as well as the CEC-Hui. When additional expertise was required, external reviewers were identified from the RCMI Coordinating Center (RCMI-CC) national database of reviewers, comprising more than 200 subject-matter experts, the vast majority with NIH study section experience. The same criteria and scoring rubric were used to evaluate full proposals. Impact scores and summary statements were evaluated by the core leaders who made funding recommendations to the Ola HAWAII Multiple Principal Investigators (MPIs). The Ola HAWAII MPIs made all final funding decisions.

### 2.3. Personalizing the Mentoring Bootcamp

A unique aspect of the Ola HAWAII IDC was the Mentoring Bootcamp. This training opportunity was open to a wide range of Hawai‘i investigators, and to external RCMI partners via livestream virtual sessions. Held in two-hour sessions three days a week, for four weeks each summer, the Ola HAWAII Mentoring Bootcamp was heavily promoted through email, newsletters, webinars, and presentations at department meetings. Flyers were sent via e-mail to deans, directors, chairs, and faculty across the 10-campus University of Hawai‘i System, and to organizations interested in forming community–academic partnerships to advance health equity, and to RCMI U54 Centers nationwide. Mentoring Bootcamp sessions covered many topics, such as grant writing, research design and project management, ethics, implementation, dissemination, IDPs, time management; topics were chosen to enrich the professional development of new and early-stage investigators. Subsequent monthly workshops were offered over the academic year to support the pool of PPP awardees, including topics such as communicating with NIH officials, FDA applications, leadership and peer-reviewing skills, and funding opportunities outside of NIH.

### 2.4. Expanding the Mentorship and Reviewers Network

Essential to the professional development of PPP investigators were expanding access to mentors and reviewers outside of Hawai‘i and linking PPP investigators, where appropriate, to other RCMI sites for training programs, multi-site projects, shared resources, and new team-science opportunities. Investigators were encouraged to access mentoring networks comprising senior faculty, qualified peers, and others who could serve as teachers, advisors, advocates, role models, and coaches. To maximize mentor–mentee relationships, sample agreements and published materials were provided to guide investigators and their research partners to articulate and document a formal agreement, defining roles and responsibilities to include specific expectations for meetings, feedback, follow-up, and closure. Ola HAWAII collaborated with the RCMI-CC and the National Research Mentoring Network (NRMN) for mentoring and investigator network development.

## 3. Results

During 2017–2022, we received 102 PPP preproposals across the three research areas of basic biomedical, clinical, and behavioral science. Of these, 45 (48%) were invited to submit full proposals, and 22 (19%) were awarded, of which eight were basic biomedical, seven were clinical, and seven were behavioral research projects. These 22 projects engaged 32 new and early-stage investigators, 18 (83%) of whom were early-career faculty (assistant professor). Most investigators were ethnic minorities and/or women (Table 1). Eighteen (83%) of the twenty-two funded pilot projects were focused on ethnic minority populations, with specific attention on NH, OPI, and Filipinos. Investigators represented different colleges/schools/departments, including Medicine (basic science and clinical disciplines), Social Work, Public Health, Nursing, Cancer Center, Pharmacy, and Natural Sciences. In addition, most pilot projects included a community collaborator with a vested interest in the integrity and relevance of the research for their communities. There is considerable overlap in investigator characteristics so that the Center/College/School appointments do not equal to the projects funded.

Extramural funding and publication data are shown in Table 2, along with the return on investment (ROI) in terms of additional extramural funding garnered by PPP awardees. Productivity is directly related to the interval between completion of the pilot project and subsequent opportunities for success with grants and publications. PPP awardees published 77 peer-reviewed manuscripts and subsequently authored competitive grants to the NIH, Robert Wood Johnson Foundation, and local funding agencies. Institutional funds were used to support additional PPP investigations during several award cycles. Awards were for one year but could be extended with Ola HAWAII leadership approval into the subsequent year using carry-forward support (e.g., when subject enrollment was delayed). From 5 to 11 investigators were supported by PPP awards each year.

Ola HAWAII cores contributed to the success of PPP awardees. Robust evaluations showed these new and early-stage investigators benefitted from the Mentoring Bootcamp, which offered diverse topics and transitioned to a virtual platform that expanded the audience base to include investigators from other RCMI U54 Centers. The RIC supported the pilot projects through consultations, technical services, and training from a variety of sub-cores, including biostatistics, bioinformatics, histopathology, behavioral, clinical research, and regulatory and compliance. Although the Ola HAWAII RIC processed more than 1300 requests for resources in this five-year period from the larger Hawai‘i research community, PPP awardees were designated the highest priority for support. The CEC and their advisory board of 10 dedicated community members guided community-engaged research in many ways, including participation in the review/funding recommendations of all PPP applications, providing direct feedback on community concerns and priorities, and supporting dissemination of research findings back to communities through community-hosted/community-placed events.

There were challenges associated with the COVID-19 pandemic that warrant comment. One challenge relates to the reduction in the number of pilot project preproposals in years 3 through 5. There was a decline in preproposals from 43 in Year 1 to 11 in Year 5. The number of full proposals fell from 18 to 5 during that time frame. The number of awarded projects ranged from three to seven per year.

Another challenge originating from the COVID-19 pandemic was meeting restrictions eliminating most in-person trainings and meetings. For PPP investigators with requisite mentoring and training needs, this necessitated a significant modification of the delivery of infrastructure services from an in-person format to an interactive virtual platform. For example, the Mentoring Bootcamp was redesigned from an in-person training program to a fully online training program offered through Zoom. A positive outcome of this change was that we were able to expand our audience to include investigators from other RCMI U54 Centers, growing from 19 attendees in 2017 to 112 in 2022. Another challenge has been the insufficient time for new and early-stage investigators to develop community ties.

### Examples of Pilot Projects and Their Effects on Community Participants

MALAMA: Backyard Aquaponics to Promote Healthy Eating and Reduce Cardiometabolic Risk (feasibility study): Jane Chung-Do (Office of Public Health Studies) and Ilima Ho-Lastimosa (College of Tropical Agriculture & Human Resources) received pilot funds to systematically test the acceptability and feasibility of backyard aquaponics workshops as a health intervention [27,28]. Using a single group pre-post design, 10 NH families (n = 21 individual participants) from Waimānalo participated in a health promotion intervention, named MALAMA (Mini Ahupuaa for Lifestyle And Meaai [food] through Aquaponics). In addition to being an acronym, the word “malama” in Hawaiian means to “take care”, “preserve”, “protect”, and “nurture.” The PPP awardees developed and implemented six family-based workshops over three months. Individuals from the community who had prior experience in aquaponics from the previous years served as Lima Kokua, or peer leaders, by sharing their lessons learned with the participants and providing support throughout the workshops. There was consistent attendance through the six workshops. Although this was a feasibility study, favorable changes in health behaviors and outcomes were found. Fruit consumption significantly increased from 2.1 to 2.9 servings/day, and favorable changes were reported in vegetable and fish consumption. There were also financial savings to growing their own fresh fruits, vegetables, and fish. A trend in reduction of systolic and diastoic blood pressure was also noted. Participants found workshops to be culturally acceptable, identified the relationship building aspects of the intervention as essential, and recommended the intervention be extended from three to six months.

The Community-Driven Approach to Mitigate COVID-19 Disparities in Hawai’i’s Vulnerable Populations: May Okihiro (Department of Pediatrics), Ruben Juarez (Department of Economics) and Alika Maunakea (Department of Anatomy, Biochemistry & Physiology) received pilot funds to assess COVID-19 vaccine hesitancy among vulnerable adults living in Hawai‘i [29]. The investigators assembled a multidisciplinary team of academic and community investigators, along with long-standing community partners across Hawai‘i, to form a collaborative called the Pacific Alliance Against COVID-19 (PAAC) to participate in the National Institutes of Health Rapid Acceleration of Diagnostics-Underserved Populations (RADx-UP) Initiative [30]. Partners included the Accountable Healthcare Alliance of Rural Oahu (AHARO), a consortium of five federally qualified community health centers (FQHCs), and K–12 public schools (kindergarten through grade 12) that serve communities on three islands with large proportions of NH and OPI. The study found that 1124 adults residing in a region with one of the lowest COVID-19 vaccination rates in Hawai‘i revealed that race/ethnicity was not directly associated with the probability of vaccine uptake. Instead, a higher degree of trust in official sources of COVID-19 information increased the probability of vaccination by 20.7%, whereas a higher trust in unofficial sources decreased the probability of vaccination by 12.5% per unit of trust [31]. These results revealed a dual and opposing role of trust on COVID-19 vaccine uptake. Interestingly, NH and OPI were the only racial/ethnic group to exhibit a significant positive association between trust in and consumption of unofficial sources of COVID-19 information, which explained the vaccine hesitancy observed in this indigenous population. These results offer novel insight relevant to COVID-19 mitigation efforts in ethnic minority populations. Behavioral and health changes in the wider community and population are difficult to measure, given these were pilot projects.

## 4. Discussion

The Ola HAWAII PPP has been highly successful in mentoring and supporting a diverse health disparities research workforce. Our challenges have provided important lessons and guided needed systemic changes. New and early-stage investigators who aspire to conduct health disparities research must also understand the issues faced by underserved communities and engage the communities as partners in identifying the causes of health disparities, devising and designing appropriate measures and methods for study, and collaboratively developing and implementing effective and culturally appropriate interventions for reducing health disparities. They also must learn to engage policymakers who can help to institutionalize evidence-based practices [32]. PPP awardees also benefitted from continuing assistance with project implementation, data analysis and data management, dissemination, time management, and research career planning. Such investigator development requires structures to monitor progress and encourage regular interaction with qualified mentors [33,34]. As suggested above, during the current funding cycle for Ola HAWAII, we plan to further enhance our PPP by (1) strengthening team-science; (2) implementing a personalized Research Studio Program; and (3) building connectivity with other infrastructure programs offering pilot project support.

### 4.1. Strengthening Team Science

Collaborating investigators must fully commit to a team-science approach so that expertise from multiple disciplines can be brought to bear on a health issue. In our second cycle, we have designed several strategies to strengthen team science. First, we will require a community collaborator on all pilot project teams to ensure that community participation occurs at all stages of research, including conceptualization, design, planning, and implementation, and that all Ola HAWAII-funded research is relevant to community concerns and sensitive to socio-cultural issues. Second, the Mentoring Bootcamp will be diversified and tailored to respond to the needs of basic biomedical, clinical, and behavioral pilot project investigators more fully. This will include selective sessions with mixed and indigenous methods expected to appeal more to clinical and behavioral researchers, while offering other selective sessions on genetic, epigenetic, and genomic analyses expected to appeal more to clinical, translational, and basic biomedical researchers. Third, Ola HAWAII will promote team-science projects with health system and community-based collaborators through recently established academic affiliation agreements with these institutions. This approach will provide an opportunity to increase the number of PPP applicants outside of the traditional university environment. Finally, while we will continue to disseminate findings to the scientific and lay communities, we will also relay critical information to policymakers who may help us to develop policies that realize effective interventions to reduce disparities.

### 4.2. Implementing a Research Studio Program

In collaboration with the CEC and IDC, the Research Capacity Core (RCC, formerly the RIC) will implement, market, and expand a personalized Research Studio Program to support PPP awardees and other investigators. Modeled after a program originally developed by the Vanderbilt Institute for Clinical and Translational Research [35] and adopted by other Clinical and Translational Science Awards (CTSA) sites [36], the Ola HAWAII Research Studio Program is designed to improve research methodology and execution through investigator-requested consultations with personalized panels of senior investigators and subject-matter experts. Each studio will consist of a 60 min session between the investigator and assembled panel, with the discussion being highly focused on one of ten components of a research project [37,38] that will be pre-specified at the investigator’s request. All relevant materials for the consultation will be provided by the investigator prior to the session to allow thoughtful review.

Three categories of research studios will be offered: (1) research development; (2) research implementation; and (3) research outcomes. The development studios will be focused on early-stage investigators to assist them in identification of mentors and collaborators, and research resources, and in the development of study hypotheses, specific aims, and overall study design. Implementation studios will focus on project implementation and monitoring, as well as data analysis and interpretation. Finally, the outcomes studios will provide specific feedback on new or resubmitted manuscripts and grant applications through a peer-review process analogous to those performed by journal reviewers and NIH study sections. Each Studio panel will include a senior investigator with grant-writing expertise and an NIH funding record, a content expert in the investigator’s research area, a data science expert, a CEC representative, and other consultants, as deemed appropriate for the project.

### 4.3. Building Connectivity with Other Infrastructure Programs Offering Pilot Project Funding

Building on our successful PPP, we will expand and enhance research partnerships with other infrastructure grants and collaborating centers that offer pilot funding. By fostering opportunities to connect with mentors and collaborators locally and nationally, we will leverage resources and accelerate research independence of new and early-stage investigators and postdoctoral fellows, particularly those from underrepresented minority groups. We plan to leverage the mentoring and professional development infrastructure offered by the RCMI-CC Clinical Research Pilot Projects Program and other NIH-supported faculty development programs at our institution. For example, we have newly partnered with the Center for Pacific Innovations, Knowledge and Opportunities (PIKO), supported by the NIGMS Institutional Development Award Networks of Clinical and Translational Research (IDeA-CTR), to avoid duplication of services and maximize resources to PPP investigators. PIKO’s pilot project investigators will have access to the Ola HAWAII Mentoring Bootcamp, and Ola HAWAII PPP investigators will have access to the PIKO five-month program of intensive, structured learning for preparation and submission of NIH grant proposals modeled after NRMN.

### 4.4. Response to the COVID-19 Pandemic

The COVID-19 pandemic reduced state revenue, which resulted in institutional financial hardships and hiring freezes. The resultant reduction in the pool of early-stage investigators significantly impacted the number of PPP preproposals towards the end of the five-year grant period. Further, application submissions from existing early-career faculty declined due to increased clinical demands during the pandemic, increased teaching demands from required curriculum virtual transitions and faculty attrition, and general difficulties with maintaining research progress during lab shutdowns, staff quarantines, and supply shortages. We are addressing these challenges in our current cycle with vigorous interactive webinar promotions, an increase in faculty hires, and through the expansion of PPP eligibility to include post-doctoral fellows and clinical residents. Individualized mentorship meetings and technical consultations between PPP investigators and Ola HAWAII cores were also managed through online platforms. Although there is an increasing return to in-person meetings, we continue to use these new technological platforms when beneficial for the PPP.

During the COVID-19 pandemic, the Ola HAWAII CEC has worked closely with more than 40 community partners to build community trust, support meaningful community–academic research partnerships, and aggressively increase community participation in basic biomedical, behavioral, and clinical research. To address this challenge in our next cycle, we are undertaking several new actions, including (1) requiring pilot project investigators to include a community collaborator with each project, and (2) implementing a community-engaged research studio program through which members from the community can provide investigators with direct feedback on the relevance of their proposed research to the community, and whether planned research approaches are respectful and culturally appropriate.

## 5. Conclusions

Our PPP awardees have been highly successful, as evidenced by subsequent extramural funding and publications, and a solid ROI. Advancing health disparities research requires a diverse scientific workforce that will drive our understanding of the causes of health disparities and lead to interventions to reduce these disparities. While Ola HAWAII has helped promising faculty members advance their careers toward research independence, the highly competitive NIH funding climate necessitates renewed efforts to support new and early-stage investigators, particularly those from underrepresented backgrounds and minority groups. Ongoing enhancement of the PPP using innovations outlined in this paper are necessary to attract and retain investigators who are pursuing a career in health disparities research.

## Figures and Tables

**Table 1:** Characteristics of Funded Pilot Projects.

Projects Funded	Lead Center/College/School	Research Category	Research Team	
22	Cancer Center (5), Engineering (1), Medical School—basic science (8), Natural Sciences (1), Nursing (1), Nutrition (1), Other Health Science (2), Pharmacy (2), Social Work & Public Health (7)	7-Clinical 7-Behavioral 8-Basic Science	N = 32 Researchers	
			10 Minority Researchers (NH *, Filipino, Black)	23 Women Researchers

* NH = Native Hawaiians.

**Table 2:** Productivity of PPP Awardees.

Project Period	Number of PPP Awards	Number of Publications	Grants Submitted	Grants Awarded	Extramural Grants Awarded	Ola HAWAII Funds	Institutional Funds	Total Pilot Funds	Return on Investment
2017–2018	7	27	25	16	$5,403,066	$270,375	$70,000	$340,375	15.8
2018–2019	5	39	15	8	$1,260,688	$231,750	$100,000	$331,750	3.8
2019–2020	3	8	37	4	$1,166,773	$231,750	$0	$231,750	5.0
2020–2021	3	1	7	3	$477,153	$231,750	$0	$231,750	2.1
2021–2022	4	2	0	0	$0	$231,750	$50,000	$281,750	0
TOTAL	22	77	84	31	$8,307,680	$1,197,375	$220,000	$1,417,375	5.9

## Data Availability

The authors will make data available directly to readers upon request.
